# Variable Colonization after Reciprocal Fecal Microbiota Transfer between Mice with Low and High Richness Microbiota

**DOI:** 10.3389/fmicb.2017.00196

**Published:** 2017-02-23

**Authors:** Aaron C. Ericsson, Alexa R. Personett, Giedre Turner, Rebecca A. Dorfmeyer, Craig L. Franklin

**Affiliations:** ^1^University of Missouri Metagenomics Center, University of MissouriColumbia, MO, USA; ^2^University of Missouri Mutant Mouse Resource and Research Center, University of MissouriColumbia, MO, USA; ^3^Comparative Metagenomics Laboratory, Department of Veterinary Pathobiology, University of MissouriColumbia, MO, USA

**Keywords:** fecal microbiota transfer, 16S rRNA gene, mouse, microbiome, richness, colonization resistance

## Abstract

Several associations have been made between characteristics of the resident gut microbiota and human health and disease susceptibility. Animal models provide the means to test these correlations prospectively and evaluate causality. Experimental fecal microbiota transfer (FMT), or the intentional transplantation of gut microbes into recipient mice depleted of their autochthonous microbes with antibiotics, is a commonly used method of testing these relationships. The true completeness of microbial transfer through such procedures is poorly documented in the literature, particularly in the context of reciprocal transfer of microbes between recipient and donor mice harboring microbial populations of differing richness and diversity. Moreover, it is unclear whether the use of frozen fecal contents or cecal contents would confer any difference in the outcomes of transfer. Herein, groups of mice colonized with distinct gut microbiota of differing richness and composition were used in a reciprocal FMT study, with different groups receiving transfer of material prepared from fresh cecal contents, fresh feces, or frozen feces. Targeted 16S rRNA gene amplicon sequencing was used at intervals throughout the study to characterize the microbiota. Notably, despite comparable depletion of the microbiota in recipient mice prior to transfer, donor-specific taxa reliably colonized recipients only when relatively rich donor material was transferred to mice originally colonized with a simpler microbiota. It is unclear whether these differences were due to differences in the endogenous recipient microbiota or host factors induced in early life by microbial factors. These findings are of practical import for researchers using FMT to prospectively assess the influence of the gut microbiota in mouse models, and to those studying host-microbial interactions and their influence on gut barrier function.

## Introduction

While it has long been appreciated that the microorganisms present in the gastrointestinal tract (GIT) of free-living animals can affect host health both positively and negatively, it is only recently that molecular methods have been developed to allow comprehensive characterization of these communities. Whole genome and targeted sequencing approaches have revealed microbial ecosystems within the gut, referred to here as the gut microbiota (GM), which outnumber host somatic cells by an order of magnitude. Impressively, the number of distinct genes encoded by the GM, collectively referred to as the gut microbiome, exceeds that of the host by greater than two orders of magnitude. With these comparisons in mind, it may not be surprising that a constant stream of data demonstrates correlations between the composition or gene content of the GM and diseases or conditions affecting not just the GIT (de Vos and de Vos, 2012), but also the cardiovascular (Tang and Hazen, 2014), endocrine (Clarke et al., 2014), and central nervous systems (Cryan and Dinan, 2012). However, the majority of data generated in human studies provide correlative associations and there is a need for robust and reproducible model systems in which causality can be tested. Rodent models are one attractive approach due to the ability to control for genetics and environmental factors, many of which can influence the composition or gene expression of the GM. Moreover, rodent models allow for longitudinal studies wherein disease can be induced by well-characterized and uniform triggers.

Experimental manipulation of the GM in rodents can be performed several different ways (Ericsson and Franklin, 2015), including rederivation via embryo transfer to germ-free status, rederivation into surrogate hosts harboring defined GM profiles, co-housing to allow transmission of gut microbes via coprophagy, and direct inoculation with pure cultures or complex mixtures of bacteria, the latter referred to as fecal microbiota transfer (FMT). FMT has gained well-deserved attention in human medicine as an effective means of treating antibiotic-induced overgrowth of *Clostridium difficile*, with an estimated therapeutic efficacy of 80–90% (Kassam et al., 2013; Kelly et al., 2014; Youngster et al., 2014a). In this context, FMT recipients are already subject to significantly decreased microbial diversity (Chang et al., 2008), typically owing to antibiotic administration prior to the proliferation of *C. difficile*. With experimental FMT using animal models, the pre-existing GM provides colonization resistance against the transferred microbiota and a cocktail of antibiotics is typically administered to animals prior to FMT to minimize this. While multiple antibiotic regimens have been used for this purpose, a cocktail comprising ampicillin, vancomycin, neomycin, and metronidazole has gained favor due to its activity against Gram positive, Gram negative, and anaerobic bacteria, as well as its potential oral administration in drinking water.

Of note however, the depletion of autochthonous bacteria following antibiotic administration is often assessed via standard microbiological culture in aerobic and anaerobic conditions (Rakoff-Nahoum et al., 2004; Turer et al., 2008; Ochoa-Repáraz et al., 2009; Kirkland et al., 2012; Ramanan et al., 2014; Knoop et al., 2016) or is not evaluated at all (Nemoto et al., 2009; Koike et al., 2012; Lee et al., 2014). Considering the recalcitrance of many gut microbes to cultivation *ex vivo*, the true efficacy of the aforementioned antibiotic cocktail in depleting the GM is unclear although one study focusing on this question indicated that a 7 day exposure was not sufficient to sterilize the gut (Croswell et al., 2009). Subsequent studies using molecular methods to assess the post-antibiotic GM in mice have reported similarly incomplete depletion of commensal gut microbes (Ichinohe et al., 2011; Dapito et al., 2012; Baldridge et al., 2015; He et al., 2015; Schuijt et al., 2016). As an extension of this, the degree to which FMT results in the successful colonization of recipient animals by donor microbes may also vary. Moreover, scant empirical data are available comparing the efficacy of frozen fecal material or fresh cecal contents in FMT. While the use of frozen feces in FMT would allow for storage and greater flexibility in experimental procedures, it is possible that freezing may induce preferential lysis of certain taxa. Alternatively, cecal contents may represent a preferable source of FMT material due to its function as a relatively protected nidus of the colonic microbiota in rodents.

As more laboratories begin to treat the GM as the independent variable using methods such as administration of antibiotics and FMT, it will be important to understand the degree to which these methods result in the removal of the endogenous GM and repopulation of the GIT with the transplanted GM. Additionally, there are well-characterized differences in the richness, diversity, and composition of the GM of mice purchased from different commercial sources (Ericsson et al., 2015), and the influence of these differences is also of interest. That is, can FMT fully reconstitute a mouse that begins with a relatively sparse GM with a richer one and, conversely, can a mouse with a relatively rich GM be reconstituted via FMT with a less rich GM?

To assess the ability of a commonly used antibiotic cocktail to deplete the GM and allow for the stable transfer of the GM from con-specific donor animals, reciprocal FMT was performed between two groups of age-, sex-, and genotype-matched mice harboring distinct GM profiles with significantly differing richness and diversity. Samples were collected longitudinally from all recipient animals and subjected to targeted 16S rRNA amplicon sequencing and a thorough statistical analysis. Additionally, separate groups of mice received FMT with fresh fecal, frozen fecal, or fresh cecal material and colonization of recipients by donor microbes was similarly compared.

## Methods

### Mice

All mice were housed at the Discovery Ridge vivarium at the University of Missouri, an AAALAC international-accredited institution, and all procedures were performed under the approval of the University of Missouri Institutional Animal Care and Use Committee and according to the guidelines put forth in the Guide for the Care and Use of Laboratory Animals. To serve as FMT recipients, twenty-four C57BL/6J (The Jackson Laboratory, Bar Harbor, ME) and twenty-five C57BL/6Hsd (Envigo, Indianapolis, IN) 6 week-old, female mice were purchased and housed in groups of four mice per cage, in individually ventilated cages (Thoren, Hazleton, PA), under a 14:10 light cycle. Mice were given irradiated, autoclaved 5,053 mouse chow (LabDiet, St. Louis, MO) and autoclaved, acidified water *ad libitum*. Recipient mice were placed in clean cages on the first day of antibiotic administration, on the first day of the FMT, and at 2 week intervals thereafter. To serve as donor mice, six age-matched female C57BL/6J and six C57BL/6Hsd mice were purchased, with direction to supply the mice from the same vendor facility isolators as the recipient mice. Donor mice were housed three mice per cage in conditions identical to those described above.

### Antibiotic administration

Following collection of the pre-treatment fecal samples, recipient mice were administered ampicillin (1 g/L), neomycin (1 g/L), metronidazole (1 g/L), and vancomycin (500 mg/L) in the drinking water for 5 consecutive days. Water containing antibiotics was prepared daily; water bottles were monitored to confirm consumption and mice were monitored on a daily basis for signs of dehydration although none were observed.

### Fecal microbiota transfer (FMT)

For FMT of fresh and frozen fecal material, donor mice were placed in empty autoclaved cages (no bedding) and allowed to defecate normally. Donor material was not collected on an individual mouse basis, but rather from all donor mice from each vendor concurrently in two collection cages, each containing three mice. Following collection of a minimum of twelve fecal pellets per substrain (i.e., vendor) using individual sterile toothpicks, half of the fecal pellets were placed in a sterile cryo-vial and immediately placed in a −80°C freezer (Revco UxF, Thermo Scientific, Waltham, MA) until used. The other half were promptly placed in 2 mL round-bottom tubes containing 800 μL autoclaved, filtered water (Milli-Q, EMD Millipore, Billerica, MA) and homogenized for 1 min at 30 Hz using a TissueLyser II (Qiagen, Valencia, CA). Homogenates were then passed through a 30 μm pore-size nylon filters to remove large particulate and fibrous matter. Fresh fecal slurries were then pooled and diluted to a volume of 2.5 mL to allow for transfer of 300 μL per mouse to eight recipients each day. For FMT of frozen fecal material, samples were removed from the −80°C freezer, allowed to thaw for ~10–15 min, and then processed as described above for fresh samples. For FMT of cecal material, one mouse from each substrain (i.e., vendor) was sacrificed each day and the entire cecal contents of each mouse collected into a 2 mL round-bottom tubes containing 800 μL autoclaved, filtered water and processed as described above. Thus, the cecal contents of each donor mouse was used to inoculate eight recipient mice. Inoculation was performed via gastric gavage of freshly prepared material on 3 consecutive days, beginning immediately after discontinuation of antibiotics and placement on untreated drinking water.

### Sample collection

Fecal samples for 16S rRNA amplicon sequencing were collected as described above with two exceptions. First, mice were placed individually in empty autoclaved cages and allowed to defecate. Second, fecal samples were collected directly into 2 mL round-bottom tubes containing 800 μL lysis buffer, prepared as previously described (Ericsson et al., 2015). Fecal samples were collected from all recipient mice at each time-point, i.e., prior to antibiotic administration; immediately after the full antibiotic regimen; and at 1, 2, and 4 weeks post-FMT. See Supplementary Figure S1.

### DNA extraction

DNA was extracted using a manual nucleic acid precipitation method, as previously described (Ericsson et al., 2015). Briefly, samples were placed in a 2 mL round-bottom tube containing 800 μL of lysis buffer and a sterile 0.5 cm diameter stainless steel bead, and homogenized with a TissueLyser II. Supernatant was collected, supplemented with 200 μL of 10M ammonium acetate, and allowed to incubate on ice for 10 min. Following centrifugation at 16,000 × g for 10 min at room temperature, supernatant was removed, mixed with an equivalent volume of isopropanol, and allowed to incubate on ice for 30 min. Precipitated nucleic acids were then pelleted at 16,000 × g for 15 min at 4°C, rinsed twice with 70% ethanol, resuspended in Tris-EDTA, and then purified using DNeasy kits (Qiagen) according to manufacturer's instructions. DNA yields were measured using a Qubit 2.0 fluorometer and Qubit dsDNA BR assay kits (Invitrogen) according to manufacturer's instructions.

### 16S rRNA library preparation and sequencing

All amplification and sequencing were performed at the University of Missouri DNA Core, as previously described (Hart et al., 2015). Briefly, normalized DNA was used as template to generate an amplicon library of the V4 region of the 16S rRNA gene. Amplicons were generated using single-indexed (Walters et al., 2011) universal primers (U515F/806R; Caporaso et al., 2011) flanked by Illumina adapter sequences, and the following PCR parameters: 98°C^(3 m)^ + [98°C^(15 s)^ + 50°C^(30 s)^ + 72°C^(30 s)^] × 25 cycles + 72°C^(7 m)^. Amplicons were pooled for sequencing using the Illumina MiSeq instrument and V2 chemistry with 2 × 250 bp paired-end reads.

### Informatics analysis

All trimming, assembly, binning, and annotation of contiguous sequences was performed at the University of Missouri Informatics Research Core Facility, as previously described (Hart et al., 2015). Briefly, contiguous sequences were assembled using FLASH software (Magoc and Salzberg, 2011) and removed if found to be short after trimming for a base quality below 31. Qiime v1.8 software (Kuczynski et al., 2011) was used to perform *de novo* and reference-based chimera detection and removal. Remaining contigs were assigned to operational taxonomic units (OTUs) via *de novo* OTU clustering with a 97% nucleotide identity. Selected OTUs were annotated using BLAST (Altschul et al., 1997) against the Greengenes database (DeSantis et al., 2006). Principal component analysis (PCA) of ¼ root-transformed sequence data and α-diversity indices were performed at the University of Missouri Metagenomics Center using open access Past 3.13 software (Hammer, 2016), downloaded on April 2, 2016.

### Gram staining

Freshly evacuated fecal samples were handled using sterile forceps and rolled across an unused glass microscope slide. Following brief heat fixation over an open flame, staining was performed using a commercially available Gram stain kit (Becton Dickinson), according to the manufacturer's instructions. Briefly, slides were first saturated with crystal violet followed by iodide. After decolorization with acetone, samples were counterstained with safranin and allowed to air dry. Slides were examined via light microscopy to determine whether feces of antibiotic-treated mice still contained bacterial forms.

### Statistical analysis

Differences in coverage and richness between donors were tested via *t*-test or Mann–Whitney rank sum test, depending on the normality of data. Differences in DNA yields between recipient strains and between pre- and post-antibiotic samples were tested via two-way analysis of variance (ANOVA). Differences in the incidence of depletion of fecal DNA to below the limit of detection via fluorometry were determined via *z*-test. Differences between donor and post-FMT recipient samples in richness and α-diversity were tested via ANOVA or Kruskal–Wallis ANOVA on ranks, depending on normality of data as pre-determined via Shapiro–Wilk normality test. All of the aforementioned testing was performed using SigmaPlot 12.3 (Systat Software Inc., San Jose, CA). Differences between donor and recipient at various time-points in β-diversity were tested using one-way permutational ANOVA (PERMANOVA) and analysis of similarity (ANOSIM) of ranked Bray-Curtis distances using Past 3.13 software. Uncorrected *p*-values below 0.001 were considered significant. For data generated from all samples except those collected immediately following the 5-day regimen of antibiotics, a cut-off of 20,000 high-quality reads was set as the inclusion criteria in further analysis. For data generated from those samples collected immediately following antibiotic treatment, samples yielding greater than 750 reads were included in further analysis.

### Availability of data and materials

The dataset supporting the conclusions of this article is available in the National Center for Biotechnology Information (NCBI) Sequence Read Archive (SRA) repository, BioProject ID PRJNA350534.

## Results

One of the overarching objectives of the present research was to determine the efficiency of transferring a richer GM into adult recipient mice harboring a less rich GM, and vice versa. Thus, the source of mice selected for analysis was based on earlier studies wherein mice purchased from Harlan Labs (now Envigo) were found to harbor a significantly richer gut microbiota (GM) than genetically similar mice from The Jackson Laboratory (Ericsson et al., 2015). To confirm this previously observed difference, the number of unique sequences detected in mice from each source, prior to antibiotic treatment and FMT, was compared. As the detected richness may be dependent on the sequencing coverage (i.e., total number of high-quality sequences detected per sample), this was also compared. Supporting earlier studies, a significantly greater number of unique sequences was detected in fecal samples from the C57BL/6Hsd (B6Hsd) mice relative to feces from C57BL/6J (B6J) mice (*p* < 0.001, Mann–Whitney rank sum test). Following annotation and binning of sequences into OTUs (i.e., groups of sequences sharing ≥97% nucleotide identity), a similar difference was detected with B6Hsd and B6J mice harboring a mean (±SEM) of 61 (±1.0) and 34 (±1.2) OTUs, respectively (*p* < 0.001, Mann–Whitney rank sum test; Supplementary Figure S2A). Interestingly, there was also a significant difference in coverage between groups although samples from B6J mice actually yielded higher numbers of high-quality sequences than samples from B6Hsd mice (*p* < 0.001, *t*-test), indicating that the difference in richness could not be explained by differential coverage (Supplementary Figure S2B).

To allow colonization with transferred GM, it is necessary to minimize colonization resistance via depletion of the endogenous GM with broad spectrum antibiotics. To assess the depletion in each substrain of mouse following 5 consecutive days of oral antibiotic administration in the drinking water, Gram stains of freshly evacuated fecal material were prepared and fecal samples were again subjected to 16S rRNA sequencing. As expected, Gram-stained fecal smears prepared from both substrains prior to antibiotic treatment revealed abundant coccoid and rod-shaped bacteria with, subjectively, a greater relative proportion of Gram-positive bacteria. Following 5 days of treatment with antibiotics in the drinking water, Gram stains revealed a dramatic reduction in bacterial load in all mice. While a few bacterial forms (both rod and coccoid) were identified in many of the Gram-stained fecal smears, the samples were largely devoid of bacteria in both groups of mice, indicating substantial, but not complete, depletion of the endogenous microbiota of both groups of recipients.

**Figure 1:**
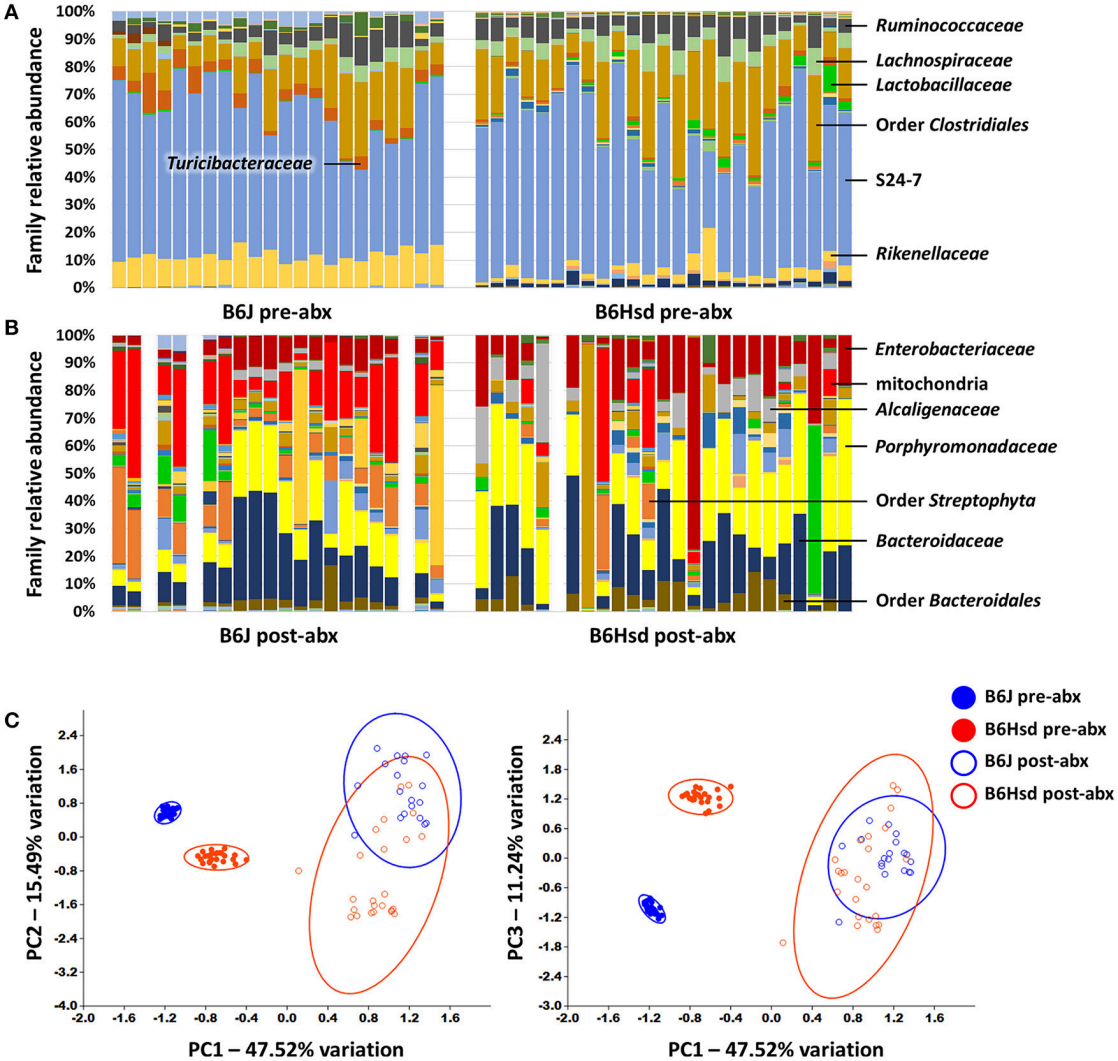
**Stacked bar charts showing the fecal microbiota detected in C57BL/6J (B6J, ***n*** = 22) and C57BL/6Hsd (B6Hsd, ***n*** =25) mice before (A)** and immediately after **(B)** 5 consecutive days of continuous exposure to broad spectrum antibiotics, as determined via 16S rRNA amplicon sequencing and annotated to the level of family. Dominant families are indicated at right or overlaid on chart and post-treatment samples returning fewer than 750 reads are indicated by a blank space. Principal component analysis of the samples shown above with shaded circles indicating 95% confidence intervals **(C)**. Operational taxonomic unit-level data were normalized via quarter root transformation. Legend at right.

To determine the composition of the GM prior to and following administration of antibiotics, extracted DNA was used as template to generate a library of 16S rRNA amplicons which were then sequenced. Prior to antibiotic treatment, the two cohorts shared the same dominant bacterial taxa, including families S24-7, *Lachnospiraceae, Ruminococcaceae, Rikenellaceae*, and order *Clostridiales*. The B6J mice also harbored substantial proportions of families *Turicibacteraceae, Anaeroplasmataceae*, and *Verrucomicrobiaceae*, while samples from the B6Hsd mice contained relatively high levels of microbes in the family *Bacteroidaceae* (Figure 1A). Following 5 days of continuous exposure to antibiotics in the drinking water, the fecal microbiota profiles all shifted dramatically to one dominated by the families *Enterobacteriaceae, Porphyromonadaceae, Alcaligenaceae, Bacteroidaceae*, and order *Bacteroidales*. Interestingly, substantial numbers of sequences were annotated to the presumably plant-origin taxa Order *Streptophyta* and family mitochondria (class *Alphaproteobacteria*, order *Rickettsiales*; Figure 1B). Despite the low DNA yields of several of these samples, all but three B6J and one B6Hsd post-antibiotic samples generated sufficient high-quality read counts to be interpreted. Taking into account all detected OTUs, pre- and post-antibiotic samples from the two groups of mice were compared using PCA (Figure 1C). As expected, the samples separated along principal component 1 (PC1; 47.51% variation) according to time-point, suggesting the greatest amount of variability in the entire dataset could be explained by treatment with antibiotics. Moreover, samples from the two groups of mice prior to treatment formed distinct clusters while there was substantial overlap of the two groups of mice post-treatment. Regardless, statistical analysis via PERMANOVA detected significant differences between all pairwise comparisons, including B6J and B6Hsd post-antibiotic samples (Table 1).

**Table 1:** **Results of PERMANOVA comparing the fecal microbiota of C57BL/6J and C57BL/6Hsd mice pre- and post-antibiotic treatment**.

		**Pre-abx C57BL/6J**	**Pre-abx C57BL/6Hsd**	**Post-abx C57BL/6J**	**Post-abx C57BL/6Hsd**
Pre-abx	C57BL/6J	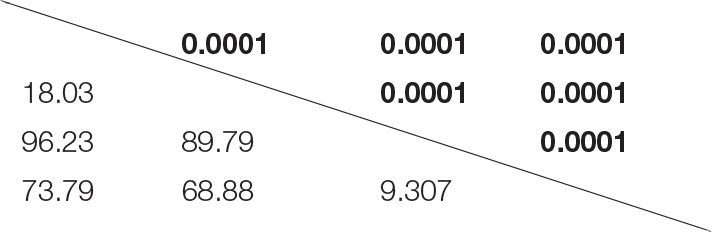			
Pre-abx	C57BL/6Hsd				
Post-abx	C57BL/6J				
Post-abx	C57BL/6Hsd				

*Values in the upper right represent p-values, with p < 0.001 in bold; values in the lower left represent F-values*.

Following antibiotic exposure for 5 days, mice were administered a slurry prepared from fresh feces, frozen feces, or fresh cecal contents by gastric gavage, prepared from donors from the reciprocal vendor, i.e., B6J mice received B6Hsd fecal or cecal material and vice versa. Recipient fecal samples collected at 1, 2, and 4 weeks post-FMT were sequenced and compared to the profiles of the donor colony. To reduce the expense and burden of analyzing the individual inocula prepared each successive day, recipient GM profiles were compared to the pre-treatment samples of the reciprocal group.

**Figure 2:**
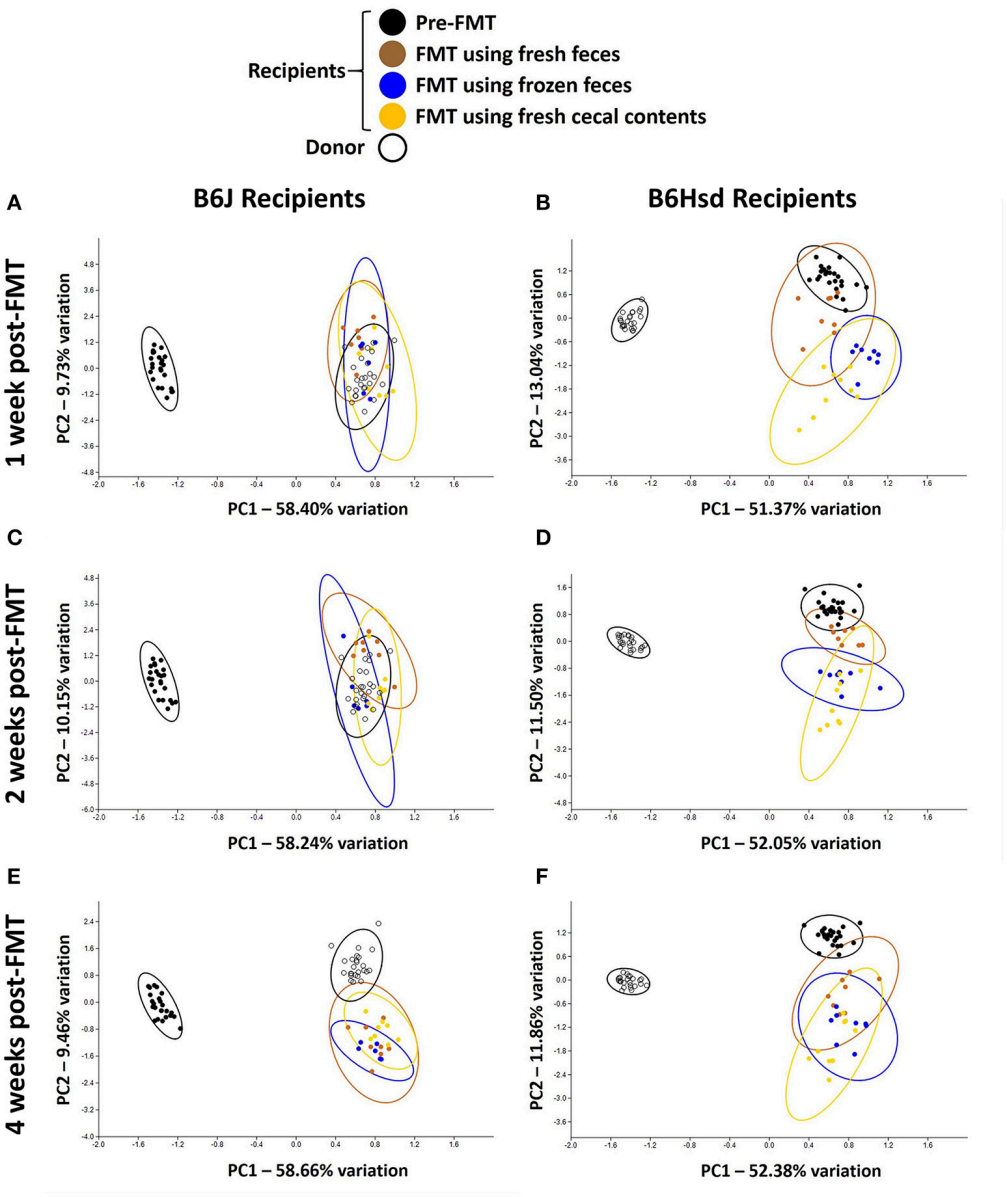
**Principal component analysis demonstrating compositional differences between fecal microbiota profiles of C57BL/6J (B6J, ***n*** = 22) and C57BL/6Hsd (B6Hsd, ***n*** = 25) recipients before fecal microbiota transfer (pre-FMT) and at 1 week (A,B)**, 2 weeks **(C,D)**, and 4 weeks **(E,F)** post-FMT with the reciprocal microbiota, legend at top.

Regarding FMT of freshly prepared B6Hsd fecal microbiota into B6J recipients, the recipient profiles were largely indistinguishable from the donors by 1 week post-FMT (Figure 2A). The profiles did not change appreciably at 2 or 4 weeks post-FMT (Figures 2C,E), suggesting that the transfer was stably introduced into the recipient mice. Multivariate analysis using PERMANOVA or ANOSIM both confirmed that while the pre-treatment B6J recipient profile differed significantly from the B6Hsd donors and most B6J recipient profiles post-FMT, the B6Hsd (donor) profile was not found to differ from the post-FMT recipient profiles (Table 2). A second objective was to determine whether frozen fecal material could be used for FMT with the same efficacy as fresh fecal material, as this would permit researchers to freeze and bank multiple samples for later use. Additionally, cecal contents were used as the source of FMT material to test whether those microbial populations might provide a more uniform inoculum. Notably, frozen fecal samples and fresh cecal contents both performed comparably to fresh feces as the sample source for FMT (Figures 2A,C,E; Table 2). Thus, the post-FMT profile resembled the donors much more closely than the pre-FMT recipient profiles, suggesting that the B6J mice were successfully inoculated with the fecal microbiota of the B6Hsd donors in all experimental groups.

**Table 2:** **Results of PERMANOVA and ANOSIM comparing the fecal microbiota of C57BL/6Hsd donors or C57BL/6J recipients prior to fecal microbiota transfer (FMT) and the same C57BL/6J recipients 1 week (1 w), 2 weeks (2 w), and 4 weeks (4 w) post-FMT using fresh feces, frozen feces, or fresh cecal contents as the source material for FMT**.

**Comparison**		**PERMANOVA**		**ANOSIM**	
		***p*****-values**	***F*****-values**	***p*****-values**	***R*****-values**
**B6/Hsd DONORS AND B6/J RECIPIENTS**					
Donor feces (*n* = 25)	Recipient feces 1 w post-FMT using fresh feces (*n* = 8)	0.0015	9.53	0.0249	0.202
	Recipient feces 2 w post-FMT using fresh feces	0.003	7.64	0.0146	0.2091
	Recipient feces 4 w post-FMT using fresh feces	0.0041	6.204	**0.0006**	0.3327
Donor feces (*n* = 25)	Recipient feces 1 w post-FMT using frozen feces (*n* = 6)	0.3974	0.8281	0.1372	0.1093
	Recipient feces 2 w post-FMT using frozen feces	0.0665	3.066	0.0176	0.2295
	Recipient feces 4 w post-FMT using frozen feces	0.0056	5.975	0.0042	0.3335
Donor feces (*n* = 25)	Recipient feces 1 w post-FMT using cecal contents (*n* = 8)	0.1296	2.001	0.1905	0.07034
	Recipient feces 2 w post-FMT using cecal contents	0.0574	2.96	0.1226	0.1098
	Recipient feces 4 w post-FMT using cecal contents	0.0228	4.158	0.019	0.2051
Recipient feces pre-FMT(*n* = 22)	Recipient feces 1 w post-FMT using fresh feces (*n* = 8)	**0.0001**	14.31	**0.0005**	0.4912
	Recipient feces 2 w post-FMT using fresh feces	**0.0001**	13.89	**0.0003**	0.5214
	Recipient feces 4 w post-FMT using fresh feces	**0.0002**	20.53	**0.0001**	0.7167
Recipient feces pre-FMT (*n* = 22)	Recipient feces 1 w post-FMT using frozen feces (*n* = 6)	0.0019	8.308	**0.0002**	0.5629
	Recipient feces 2 w post-FMT using frozen feces	**0.0007**	12.31	**0.0001**	0.6443
	Recipient feces 4 w post-FMT using frozen feces	**0.0002**	16.55	**0.0001**	0.6932
Recipient feces pre-FMT (*n* = 22)	Recipient feces 1 w post-FMT using cecal contents (*n* = 8)	**0.0002**	12.4	**0.0001**	0.5983
	Recipient feces 2 w post-FMT using cecal contents	**0.0001**	15.77	**0.0001**	0.6337
	Recipient feces 4 w post-FMT using cecal contents	**0.0001**	16.48	**0.0001**	0.6046

*Significant p-values indicated in bold*.

Conversely however, when the B6J donor colony was compared to B6Hsd recipients before and after FMT, the pre-FMT profiles changed minimally following the procedure (Figures 2B,D,F). Testing via PERMANOVA and ANOSIM confirmed that the B6Hsd recipient profiles differed significantly at all time points post-FMT from the B6J donors (Table 3). When B6Hsd post-FMT profiles were compared to the pre-existing fecal microbiota of those colonies, no differences were detected among any of the mice that received FMT prepared from fresh B6J feces, or at 1 and 2 weeks post-FMT in mice receiving FMT prepared from frozen B6J feces. Interestingly, there was a significant difference between the pre-FMT profile and all post-FMT time-points in B6Hsd recipients receiving B6J cecal contents (Table 3). Taken collectively, the above data suggest that the transfer of fecal microbiota from mice harboring the richer B6Hsd GM into recipients originally colonized with the simpler B6J GM was largely successful, while the reciprocal procedure transferring a simpler GM into mice originally colonized with a richer GM was much less efficacious.

**Table 3:** **Results of PERMANOVA and ANOSIM comparing the fecal microbiota of C57BL/6J donors or C57BL/6Hsd recipients prior to fecal microbiota transfer (FMT) and the same C57BL/6Hsd recipients 1 week (1 w), 2 weeks (2 w), and 4 weeks (4 w) post-FMT using fresh feces, frozen feces, or fresh cecal contents as the source material for FMT**.

		**PERMANOVA**		**ANOSIM**	
		***p*****-values**	***F*****-values**	***p*****-values**	***R*****-values**
**B6/J DONORS AND B6/Hsd RECIPIENTS**					
Donor feces (*n* = 24)	Recipient feces 1 w post-FMT using fresh feces (*n* = 8)	**0.0004**	9.626	**0.0001**	0.5059
	Recipient feces 2 w post-FMT using fresh feces	**0.0004**	10.6	**0.0002**	0.5378
	Recipient feces 4 w post-FMT using fresh feces	**0.0001**	12.47	**0.0002**	0.5522
Donor feces (*n* = 24)	Recipient feces 1 w post-FMT using frozen feces (*n* = 8)	**0.0001**	17	**0.0001**	0.7224
	Recipient feces 2 w post-FMT using frozen feces	**0.0001**	25.23	**0.0001**	0.7361
	Recipient feces 4 w post-FMT using frozen feces	**0.0001**	22.98	**0.0001**	0.7342
Donor feces (*n* = 24)	Recipient feces 1 w post-FMT using cecal contents (*n* = 9)	**0.0001**	15.56	**0.0001**	0.6982
	Recipient feces 2 w post-FMT using cecal contents	**0.0001**	21.41	**0.0001**	0.7943
	Recipient feces 4 w post-FMT using cecal contents	**0.0001**	16.71	**0.0001**	0.7136
Recipient feces pre-FMT (*n* = 25)	Recipient feces 1 w post-FMT using fresh feces (*n* = 8)	0.1411	1.945	0.3223	0.03354
	Recipient feces 2 w post-FMT using fresh feces	0.0889	2.549	0.0832	0.1204
	Recipient feces 4 w post-FMT using fresh feces	0.0481	3.045	0.0277	0.2002
Recipient feces pre-FMT (*n* = 25)	Recipient feces 1 w post-FMT using frozen feces (*n* = 8)	0.0073	5.056	0.0035	0.2973
	Recipient feces 2 w post-FMT using frozen feces	0.0055	6.873	0.0012	0.335
	Recipient feces 4 w post-FMT using frozen feces	**0.0003**	10.71	**0.0001**	0.5049
Recipient feces pre-FMT (*n* = 25)	Recipient feces 1 w post-FMT using cecal contents (*n* = 9)	**0.0001**	10.48	**0.0001**	0.5381
	Recipient feces 2 w post-FMT using cecal contents	**0.0001**	10.14	**0.0001**	0.5573
	Recipient feces 4 w post-FMT using cecal contents	**0.0001**	8.241	**0.0001**	0.4945

*Significant p-values indicated in bold*.

**Figure 3:**
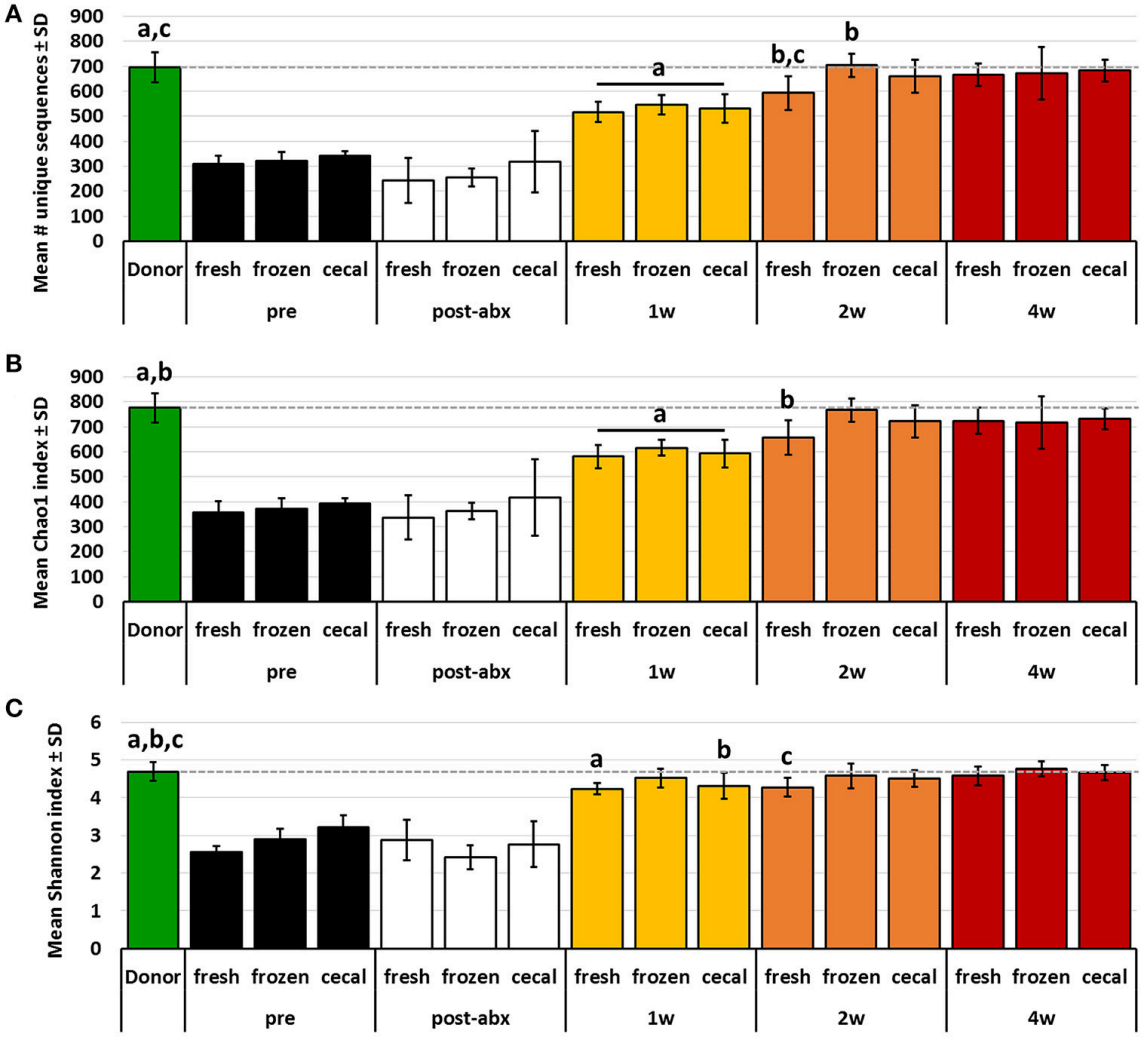
**Bar charts showing the richness (A)**, Chao1 diversity index **(B)**, and Shannon diversity index **(C)** of B6Hsd donor fecal profiles, and B6J recipients prior to any manipulation (pre), immediately after antibiotic treatment (post-abx), and at 1 week (1 w), 2 weeks (2 w), and 4 weeks (4 w) after receiving FMT prepared from fresh (*n* = 8) or frozen (*n* = 6) feces, or fresh cecal contents (*n* = 8). Dotted line indicates mean value of donor material; like letters indicate significant differences (*p* < 0.05, ANOVA). Pre- and post-antibiotic samples included for reference but not included in statistical testing.

While PCA and multivariate statistical analysis are useful for visualizing and testing for differences in the overall microbial community structure, they may not completely reflect the degree to which rare microbes were transferred during FMT. Thus, richness and diversity were compared between donor profiles and post-FMT recipients at each time-point, and the proportion of donor taxa transferred to recipients was compared between groups of mice receiving FMT prepared from the different source materials. Considering first the transfer of the relatively richer B6Hsd material into B6J recipients, both richness and diversity, as measured via number of unique sequences (Figure 3A) and Chao1 (Figure 3B) and Shannon (Figure 3C) diversity indices, were largely restored in recipients by 2 weeks post-FMT to levels comparable to those detected in donor mice. Notably, very few differences were detected in richness or diversity between recipient mice receiving FMT prepared from the different source materials at any given time-point, with the exception of greater richness at 2 weeks post-FMT in samples from mice that received FMT prepared from frozen feces relative to mice receiving FMT prepared from fresh feces.

**Figure 4:**
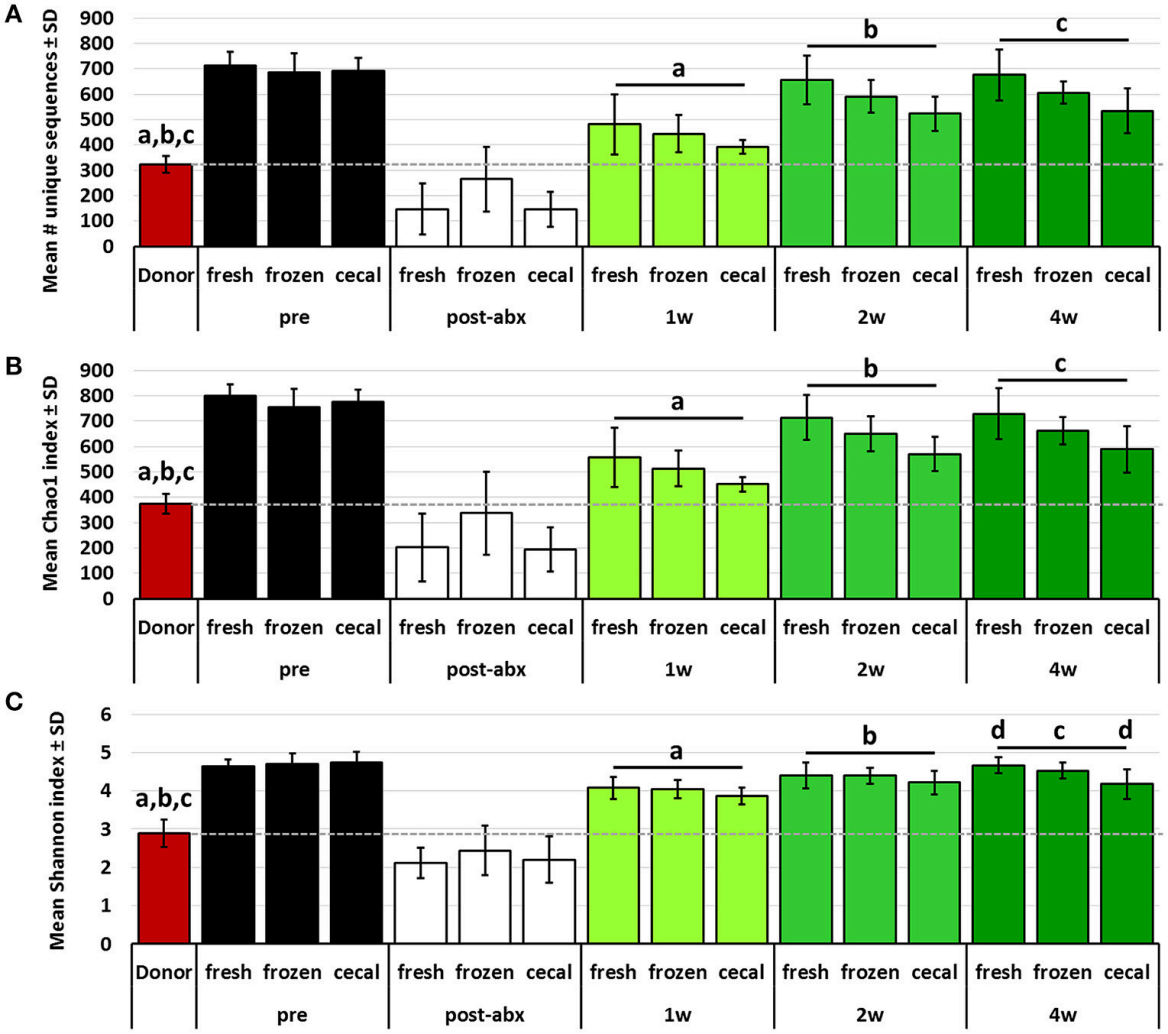
**Bar charts showing the richness (A)**, Chao1 diversity index **(B)**, and Shannon diversity index **(C)** of B6J donor fecal profiles, and B6Hsd recipients prior to any manipulation (pre), immediately after antibiotic treatment (post-abx), and at 1 week (1 w), 2 weeks (2 w), and 4 weeks (4 w) after receiving FMT prepared from fresh (*n* = 8) or frozen (*n* = 8) feces, or fresh cecal contents (*n* = 9). Dotted line indicates mean value of donor material; like letters indicate significant differences (*p* < 0.05, ANOVA). Pre- and post-antibiotic samples included for reference but not included in statistical testing.

Conversely, the richness and diversity of B6Hsd recipient profiles were significantly greater than the donor fecal profiles at all post-FMT time-points, steadily increasing from 1 to 4 weeks post-FMT and approaching the pre-FMT B6Hsd recipient levels (Figure 4). These data indicate that, despite the significant reduction of richness and diversity following antibiotic treatment to levels below that of even the donor material, all three metrics quickly rebounded to levels significantly greater than that of the donor material. Moreover, there was an apparent difference in the richness and diversity of B6Hsd recipient profiles dependent on the source of material transferred. Specifically, B6Hsd mice receiving FMT prepared from frozen feces or cecal contents demonstrated a trend toward lower richness and diversity at all time-points post-FMT than mice receiving FMT prepared from fresh feces. Considering the fact that all post-FMT time-points harbored richer and more diverse communities than the original donor material, a cause for this potential difference was unclear.

**Figure 5:**
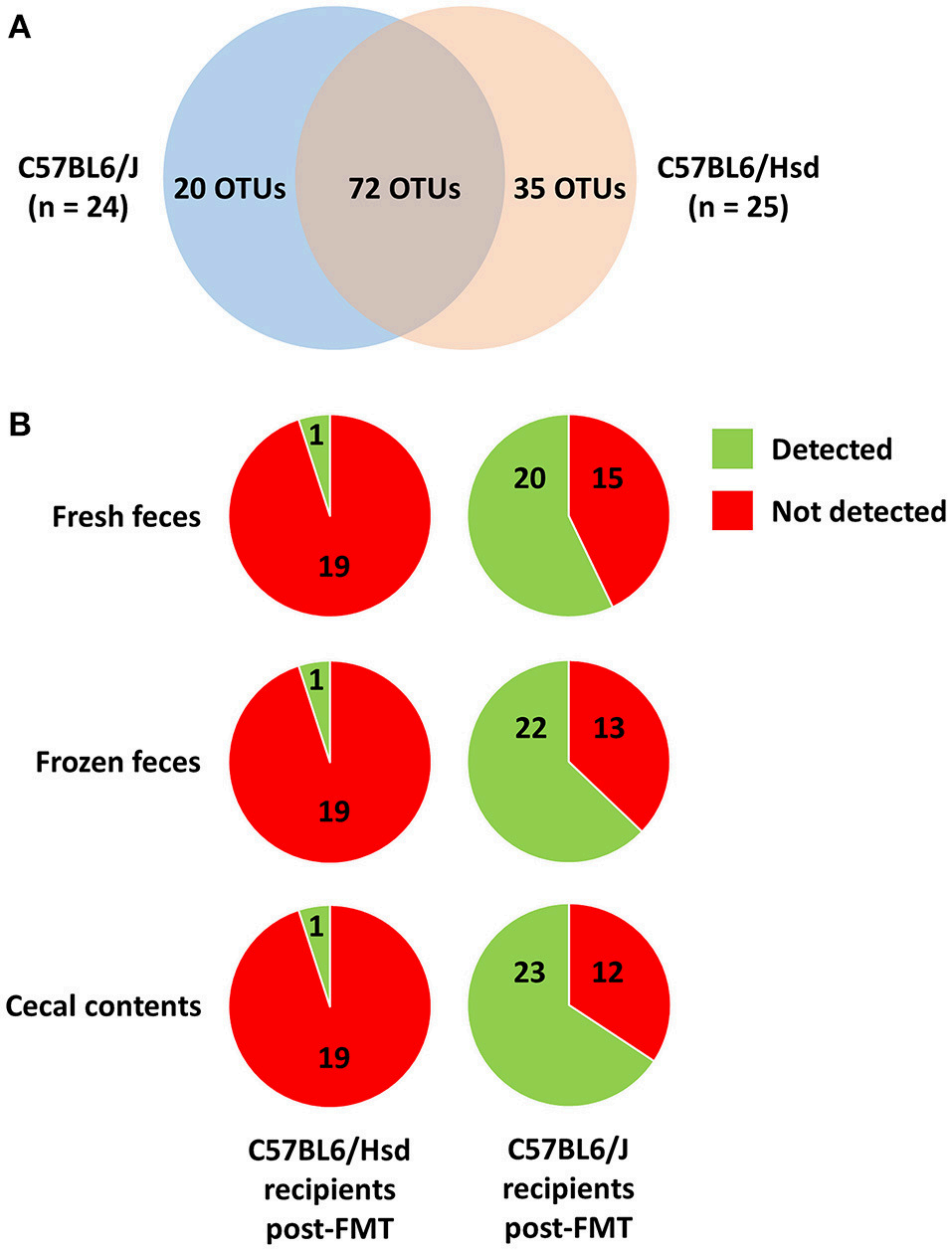
**Venn diagram showing the distribution of 127 operational taxonomic units (OTUs) detected among the C57BL/6J (***n*** = 24) and C57BL/6Hsd (***n*** = 25) donor colonies pre-FMT (A)**, and the percentage of the 20 and 35 OTUs detected exclusively in C57BL/6J and C57BL/6Hsd donors, respectively, that were detected at any time point post-FMT in the recipient mice receiving FMT prepared from fresh feces, frozen feces, or cecal contents **(B)**.

While the pre-existing B6Hsd profiles were much richer than B6J profiles, there were nonetheless many taxa unique to both microbial communities. After binning of sequences into OTUs (i.e., groups of sequences sharing a minimum of 97% nucleotide identity), a total of 127 different OTUs was initially detected across both groups, with 20 and 35 of those OTUs detected exclusively in B6J or B6Hsd mice respectively (Figure 5A). Thus, one possible explanation for the observed differences in richness and diversity between B6Hsd recipients receiving the different source materials is the relatively greater transfer of novel taxa present in fresh B6J feces relative to the other groups. However, no differences were detected in the number of novel OTUs detected in B6Hsd recipients that could be attributed to the FMT donor material and, in fact, only one novel B6J-origin OTU was detected in any of the B6Hsd recipient groups (Figure 5B). Additionally, those novel OTUs were each detected at extremely low relative abundance in only one mouse at one of the three post-FMT time-points, collectively suggesting negligible stable transfer of B6J microbiota to B6Hsd recipients, regardless of source material.

## Discussion

In summary, the data described above suggest that experimental FMT, when performed using a common antibiotic regimen to deplete the autochthonous microbiota, may only be effective in supplementing relatively sparse microbial communities with taxa from richer sources. The reciprocal procedure, i.e., FMT to re-populate mice with a less rich and diverse microbial community than was originally present, evinced negligible transfer regardless of source material or time-point analyzed post-FMT. One likely explanation for the rapid return of the fecal microbiota to a composition matching its original richness and structure is inadequate depletion of microbes with antibiotics. While Gram staining indicated that the autochthonous microbiota had been greatly reduced in both B6J and B6Hsd recipients, comparison of richness and alpha diversity indices following antibiotic administration suggested more complete depletion in the B6J recipients. Thus, the inability to stably transfer the B6J microbiota into B6Hsd recipients may be a function of incomplete removal of the pre-existing microbiota still occupying mucosal niches. It is plausible that low numbers of viable microbes remained in protected niches within the GIT (e.g., mucosal biofilms; MacFarlane and Dillon, 2007) throughout antibiotic treatment, and then rebounded following cessation. While this seems like a logical interpretation for the seeming reappearance of taxa not detected in the feces of antibiotic-treated mice, it is worth noting that despite the substantial (albeit incomplete) depletion of bacteria, very few (if any) of the B6J-specific microbes were detected in B6Hsd recipients. One possible explanation is a loss of viability of transferred B6J microbes during the preparation and administration of the FMT, although it seems highly unlikely that such a phenomenon would affect the microbes specific to the B6J GM and not the B6Hsd GM. Alternatively, the reduced microbial biomass that remained in B6Hsd recipients following antibiotic treatment was still capable of eliciting something similar to colonization resistance, or the GM originally present in the B6Hsd mice induced changes in the host that were responsible for the observed difference in transfer of detectable donor-specific taxa, independent of the antibiotic-induced GM depletion, such as upregulated IgA or defensin production. Considering that the recipient B6Hsd mice were likely originally colonized with segmented filamentous bacteria (Ericsson et al., 2015), which have been shown to promote colonization resistance against several enteric pathogens (Garland et al., 1982; Heczko et al., 2000; Ivanov et al., 2009), an indirect mechanism mediated by GM-induced changes in host intestinal gene expression is entirely plausible. The concept of colonization resistance is traditionally put forth in the context of pathogenic organisms such as enteropathogenic *E. coli* or *Salmonella enterica*, however the underlying mechanisms are non-specific and could ostensibly limit colonization by resident microbes in the transferred material. That SFB could play a role is supported by our historical dataset of endemically SFB-colonized mice from Envigo (i.e., B6Hsd mice) and a lack of SFB in mice from the Jackson Laboratory (i.e., B6J mice), and abundant data linking SFB to significantly enhanced T_H_17 profiles in intestinal lymphocytes and, by extension, production of IgA and broad-spectrum antimicrobial peptides (Buffie and Pamer, 2013). Lastly, there could be genetic differences between the B6J and B6Hsd substrains contributing to a differential receptivity to transferred microbes. Multiple groups have documented substantial genomic differences between the B6 substrains used in the current study when analyzed using high-density single-nucleotide polymorphisms (Mekada et al., 2009; Zurita et al., 2011) and larger structural variants (Yalcin et al., 2011). Moreover, substrain-dependent phenotypic differences have been reported by several groups, and it is entirely possible that such differences may have contributed to the differential colonization of recipient mice in the current study.

It should also be recognized that the antibiotic cocktail used in the current study was administered in the drinking water for 5 days, whereas others have used longer durations of exposure or daily oral gavage of the antibiotics to ensure delivery. Indeed, a review of the literature reveals several different antibiotic regimens ranging from a single administration to induce dysbiosis to 4 weeks of continuous exposure, and we opted to use a 5-day duration for two primary reasons. First, even after 3–4 weeks of continuous exposure to the same antibiotics, there is incomplete sterilization of the gut (Ichinohe et al., 2011; Dapito et al., 2012; He et al., 2015; Schuijt et al., 2016). Second, continuous long-term administration of multiple antibiotics in the drinking water becomes prohibitively expensive for larger studies while repeated twice daily oral gavage becomes time- and labor-intensive. Rather, our goal was to evaluate the feasibility of manipulating the established GM with minimal burden on the investigator. It is plausible that the transfer of the B6J GM into B6Hsd recipients may have been more complete following longer exposure to antibiotics. Considering however the possible explanations for the difference in transfer offered above (i.e., loss of viability during FMT preparation, residual microbes spared by the antibiotics, or GM-specific effects on host immunity), additional time exposed to antibiotics would not likely have increased the efficacy of FMT.

Regarding the use of frozen feces for FMT, the current data support its ability to serve as the inoculum. No significant difference was detected in the richness, diversity, or community structure in recipient mice receiving FMT prepared from frozen donor feces. One limitation of the current study however was the relatively short time samples spent in the freezer. It is possible that longer storage times at −80°C may negatively affect the viability of certain taxa for these purposes. That said, multiple reports have demonstrated comparable clinical efficacy when treating *C. difficile* overgrowth via FMT using frozen feces (Youngster et al., 2014a,b; Lee et al., 2016), providing further evidence that freezing does not have a substantial negative impact on the inoculum.

As for FMT using cecal contents, the human cecal microbiota is reported to harbor a lower abundance of anaerobes relative to the feces, while a greater proportion of those anaerobes are facultative (Marteau et al., 2001). As the cecum of rodents is a much more distinct anatomic region, we hypothesized that such differences might be even more pronounced, allowing for better survival of those microbes during preparation of the FMT. However, there was again no significant difference detected in the richness, diversity, or community composition of the fecal microbiota of mice receiving FMT prepared from cecal contents. Notably, the cecal contents of recipients were not analyzed and it is possible that the transfer of cecal microbiota did lead to differential changes in the cecum and feces of recipient mice.

In conclusion, experimental FMT using the protocol employed in the present study resulted in successful transfer of a richer GM harboring SFB into recipient mice starting with a comparatively less rich, SFB-free GM, while the reciprocal FMT resulted in negligible change in the recipient GM. The cause, or causes, for these differences are unclear but are likely associated with residual GM in the B6/Hsd recipient mice following antibiotic depletion, or a greater relative abundance of some host immunological factor involved in resistance to reconstitution which, in turn, could have been a function of differences in the pre-existing GM between the two groups. These findings highlight the need for longitudinal validation in studies using FMT, particularly in the context of no difference or change in the dependent variable following FMT. More generally speaking, the data presented here indicate that transfer of different gut microbial populations via FMT does not necessarily produce the same output in recipient mice. Additional studies will be needed to determine whether the observed differences in colonization, despite a substantial (albeit incomplete) depletion of the pre-existing GM, could be exploited as a therapeutic or prophylactic measure in humans.

## Author contributions

AE and CF conceived and designed the experiment, provided reagents and materials, and analyzed and interpreted the data. AP, GT, and RD performed the experiments, helped analyze the data, and reviewed the manuscript. AE and CF wrote the manuscript.

## Funding

Studies were performed through a grant from the NIH to the MU Mutant Mouse Resource and Research Center (MMRRC) U42 OD010918-16.

### Conflict of interest statement

The authors declare that the research was conducted in the absence of any commercial or financial relationships that could be construed as a potential conflict of interest.

## Supplementary material

Supplementary Figure S1 **Schematic representation of the experimental design and group sample sizes**.

Supplementary Figure S2 **Bar charts showing mean (± SEM) number of operational taxonomic units (OTUs) detected in fecal samples from C57BL/6J (B6J) and C57BL/6Hsd (B6Hsd) mice prior to antibiotic treatment and fecal microbiota transfer, bar indicates significant difference (*p* < 0.001) as determined by Mann–Whitney rank sum test (A);** Rarefaction of those same samples plotting total number of unique sequences detected in each sample against number of high quality reads obtained (i.e., coverage) **(B)**.
