# Supplementary figures and images for: Variable Colonization after Reciprocal Fecal Microbiota Transfer between Mice with Low and High Richness Microbiota
Source: Front Microbiol. 2017 Feb 23;8:196. doi: 10.3389/fmicb.2017.00196 (PMC5322181; doi:10.3389/fmicb.2017.00196)

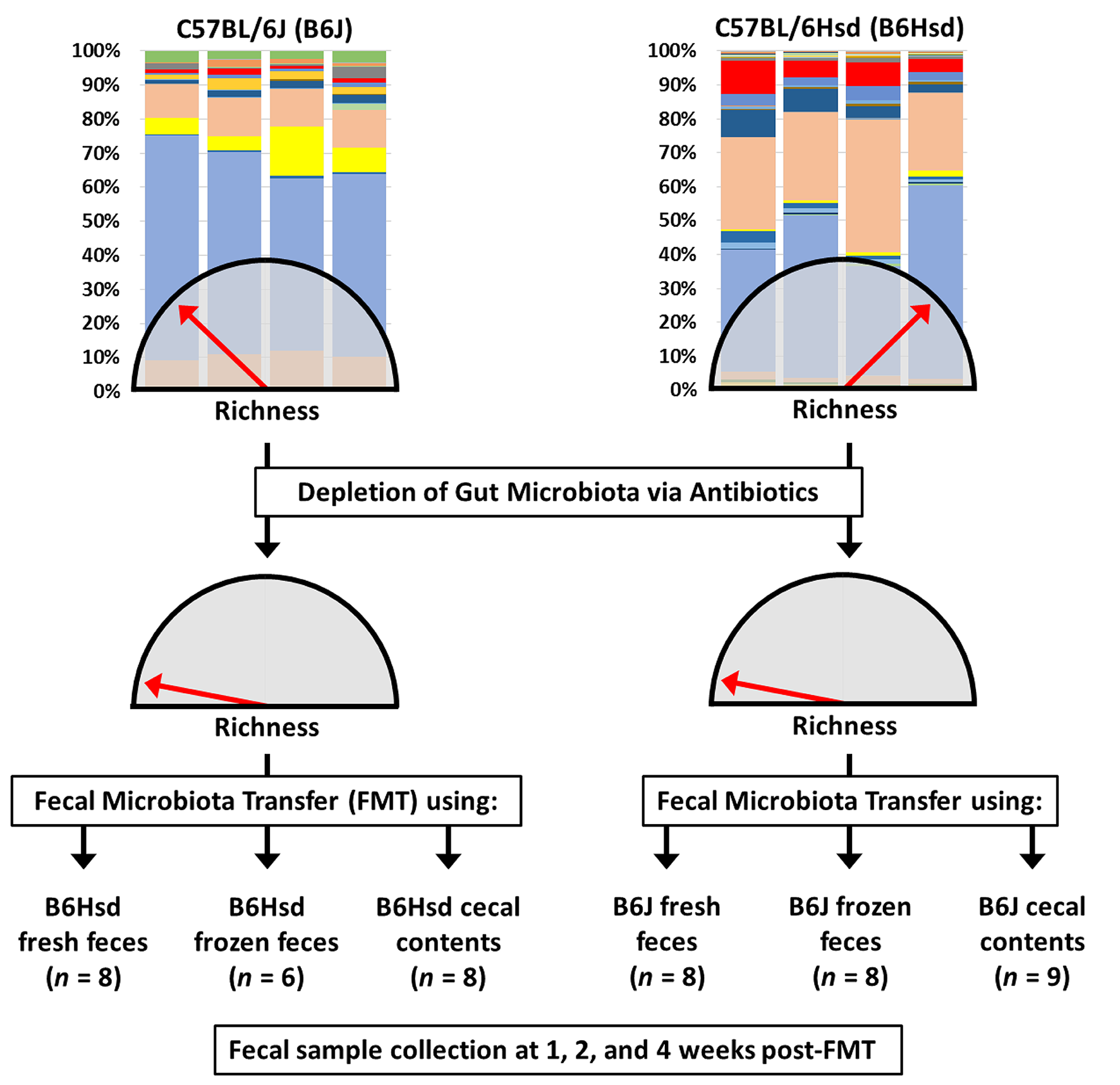

Supplement: Supplementary Figure S1 — Schematic representation of the experimental design and group sample sizes. [file Image1.TIF]

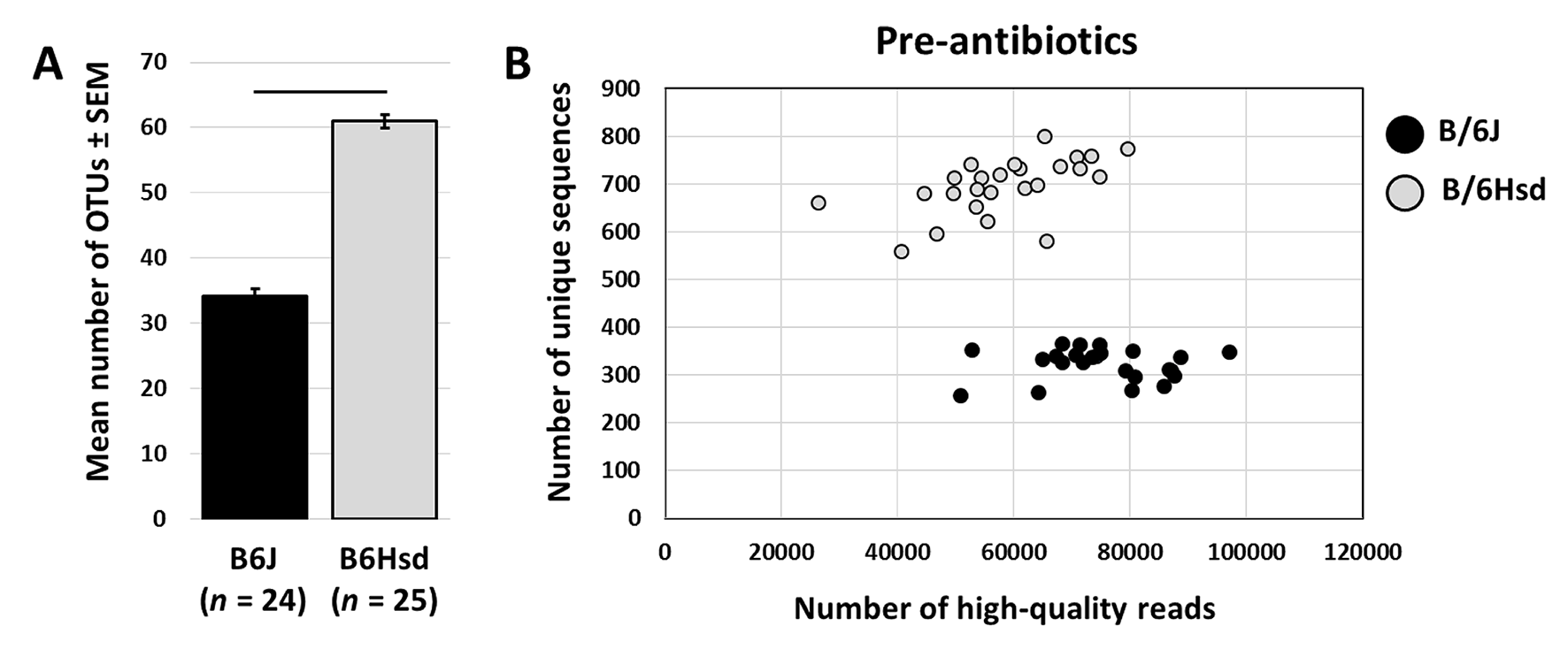

Supplement: Supplementary Figure S2 — Bar charts showing mean (± SEM) number of operational taxonomic units (OTUs) detected in fecal samples from C57BL/6J (B6J) and C57BL/6Hsd (B6Hsd) mice prior to antibiotic treatment and fecal microbiota transfer, bar indicates significant difference (p < 0.001) as determined by Mann–Whitney rank sum test (A); Rarefaction of those same samples plotting total number of unique sequences detected in each sample against number of high quality reads obtained (i.e., coverage) (B). [file Image2.TIF]
